# Spectrometric Characterization of Clinical and Environmental Isolates of *Aspergillus* Series *Versicolores*

**DOI:** 10.3390/jof9090868

**Published:** 2023-08-23

**Authors:** Océane Jomat, Antoine Géry, Astrid Leudet, Agathe Capitaine, David Garon, Julie Bonhomme

**Affiliations:** 1 Mycology and Parasitology Department, Caen University Hospital, UNICAEN, Normandie University, 14000 Caen, France; astrid.leudet@gmail.com (A.L.); capitaine-a@chu-caen.fr (A.C.); 2 ToxEMAC-ABTE, UNICAEN & UNIROUEN, Normandie University, 14000 Caen, France; antoine.gery@unicaen.fr (A.G.); david.garon@unicaen.fr (D.G.)

**Keywords:** *Aspergillus* series *Versicolores*, taxonomy, spectral clustering, spectrometric characterization, MALDI-TOF MS (Matrix-Assisted Laser Desorption/Ionization Time-Of-Flight Mass Spectrometry)

## Abstract

*Aspergillus* series *Versicolores* are molds distributed among 17 species, commonly found in our environment, and responsible for infections. Since 2022, a new taxonomy has grouped them into 4 major lineages: *A. versicolor*, *A. subversicolor*, *A. sydowii*, and *A. creber*. Matrix-Assisted Laser Desorption/Ionization Time-Of-Flight Mass Spectrometry (MALDI-TOF MS) could be a faster and more cost-effective alternative to molecular techniques for identifying them by developing a local database. To evaluate this technique, 30 isolates from *Aspergillus* series *Versicolores* were used. A total of 59 main spectra profiles (MSPs) were created in the local database. This protocol enabled accurate identification of 100% of the extracted isolates, of which 97% (29/30) were correctly identified with a log score ≥ 2.00. Some MSPs recorded as *Aspergillus versicolor* in the supplier’s database could lead to false identifications as they did not match with the correct lineages. Although the local database is still limited in the number and diversity of species of *Aspergillus* series *Versicolores*, it is sufficiently effective for correct lineage identification according to the latest taxonomic revision, and better than the MALDI-TOF MS supplier’s database. This technology could improve the speed and accuracy of routine fungal identification for these species.

## 1. Introduction

*Aspergillus* series *Versicolores* is a taxonomic group of *Aspergillus* section *Nidulantes*. These fungi are commonly found in soil [1], food [2], caves, and indoor environments with bioaerosols and moldy surfaces [3]. They have a significant impact on the quality of agricultural products, as well as on indoor air quality, and can cause health problems in humans and animals through exposure to their spores and mycotoxins such as sterigmatocystin and aflatoxins [4].

Although *Aspergillus* series *Versicolores* is not a common cause of human infections, these fungi can cause allergic reactions such as allergic bronchopulmonary aspergillosis [5,6] and lead to a variety of infections, including invasive pulmonary aspergillosis [7], fungal sinusitis [8], onychomycosis [9], and endophthalmitis [10]. These infections are generally associated with immunocompromised individuals, such as those with human immunodeficiency virus/acquired immunodeficiency syndrome (HIV/AIDS) [10] or organ transplant recipients [11]. It is therefore important to identify the exact species involved in human pathologies in order to adapt treatment [12].

*Aspergillus* series *Versicolores* has undergone several taxonomic revisions over the years. The earliest taxonomic studies of *Aspergillus versicolor* date back to the early 20th century based on morphological characteristics [13]. In 2008, Peterson introduced molecular approaches to the study of fungal systematics, leading to a more integrated approach to taxonomy that combined molecular and morphological data [14]. In more recent years, further taxonomic revisions have been carried out using a combination of morphological, molecular, and chemical data and have revealed 17 species of *Aspergillus* series *Versicolores* [15,16]. In many studies, misidentifications have been observed between *A. versicolor* species and other species of *Aspergillus* series *Versicolores* [12]. As molecular and genomic tools continue to improve, a new revision of taxonomy was proposed in 2022 and was based on a combination of phylogenetic, morphological, and molecular features, including spore morphology, growth temperature, DNA sequence analysis, and osmotic tolerance. The *Versicolores* series was reduced to four species, namely the *A. subversicolor* lineage and the *A. sydowii* lineage, each containing a single species, while the *A. versicolor* lineage contained nine species (*A. amoenus*, *A. austroafricanus*, *A. fructus*, *A. griseoaurantiacus*, *A. hongkongensis*, *A. pepii*, *A. protuberus*, *A. tabacinus*, and *A. versicolor*), and the *A. creber* lineage including six species (*A. creber*, *A. cvjetkovicii*, *A. jensenii*, *A. puulaauensis*, *A. tennesseensis*, and *A. venenatus*) [17].

Although molecular and morphological identification methods have been useful for the identification of *Aspergillus* series *Versicolores*, they have some limitations, particularly with regard to their similarity, intraspecific variability [18], and basic local alignment search tool (BLAST) similarity searches using different genes, which result in different identifications [17]. Matrix-Assisted Laser Desorption/Ionization Time-Of-Flight Mass Spectrometry (MALDI-TOF MS) has become the reference technique for the rapid identification and classification of microorganisms, including fungi [19,20,21]. Several MALDI-TOF MS systems are available worldwide, such as the Bruker Biotyper, but their database may not be able to distinguish closely related species within the *Aspergillus* series *Versicolores*. First of all, the lack of completeness of these databases and their late updates in relation to taxonomic revisions may simply explain the misidentifications. Thus, several studies have reported difficulties in identifying rare *Aspergillus* species using the Bruker database [22,23,24]. Moreover, for *Aspergillus* species of the *Versicolores* series, their high level of intraspecific variability can result in overlapping mass spectra, making it difficult to distinguish between species.

To overcome the weaknesses of the databases provided by MALDI-TOF MS suppliers, it is possible to generate local databases. Several teams have demonstrated their usefulness for correct species identification, in particular for *Achromobacter* [25], *Clostridia* [26], and lice [27].

The objective of this study was to evaluate the interest of spectrometric identification of *Aspergillus* species of the *Versicolores* series by creating a local database at the University Hospital of Caen.

## 2. Materials and Methods

### 2.1. Clinical and Environmental Isolates

The 30 isolates used to develop the local mass spectrometry local database are described in Table 1.

They were obtained from clinical samples received at the Mycology Laboratory of the Caen University Hospital (France) and from environmental samples collected by the ToxEMAC team, which has the largest French collection of *Aspergillus* isolates of the *Versicolores* series. We evaluated 8 species of the series, some of which were represented by a single isolate, such as *A. amoenus*, *A. fructus*, *A. protuberus*, *A. puulaauensis,* and *A. tabacinus*, while others were represented by multiple isolates (*A. creber*, *n* = 10; *A. jensenii*, *n* = 4; *A. sydowii*, *n* = 11). All isolates were preserved in the ToxEMAC mycological bank (stored at −80 °C).

### 2.2. Molecular Identification

All the steps leading to the molecular identification of the isolates used in this study have already been described previously [4]: collection of environmental and clinical samples, fungal culture on malt extract agar (MEA), DNA extraction, and purification using the NucleoSpin Plant II kit and NucleoSpin gDNA Clean-up kit (Macherey-Nagel, Duren, Germany), respectively, and PCR using the beta-tubulin gene (*BenA*) with Bt2a/Bt2b primers and DNA sequencing.

### 2.3. Generation of the Local Database

#### 2.3.1. Culture

The isolates were grown on Sabouraud-chloramphenicol-gentamicin agar (SAB) (ThermoFisher Scientific, Waltham, MA, USA), malt extract agar medium supplemented with 0.02% (*m*/*v*) chloramphenicol (MEA) (Cooper, Melun, France), and CZAPEK agar (CZA) (Cooper, Melun, France), and were all incubated at 30 °C for 2–14 days. The complete extraction was performed on a sterile tube containing 2 mL of Sabouraud-chloramphenicol liquid medium (Liquid SAB) (Biomérieux, Lyon, France) and was incubated at 24 h. The detection of a fungal colony was identified each time by phenotypic and MS methods. For each culture on SAB and MEA media, a first extraction was performed the day following the start of fungal growth (young colonies) and possibly repeated two days in a row (mature colonies), which was considered a first attempt. In case of failure, a new extraction was carried out in the same way after subculture on a new medium for young and mature colonies (second attempt). In case of another failure, the previous step was repeated (third attempt) only for cultures on SAB medium. Extraction tests were carried out first on young colonies, and when this worked, new attempts were made on older colonies. The objective was therefore to generate two main spectra profiles (MSPs) (from young and mature colonies) in a single culture medium attempt to assess whether there was a spectral difference between young and mature colonies considered.

#### 2.3.2. Protein Extraction

Filamentous fungi grown on the medium were collected by scraping the surface of the colonies with a sterile loop (VWR International SAS, Radnor, PA, USA). Lentil-sized fungal material (hyphae and spores) was deposited in a sterile Eppendorf tube containing 300 μL of high-pressure liquid chromatography (HPLC) water (VWR International SAS, Radnor, PA, USA) and 900 μL of absolute ethanol (VWR International SAS, Radnor, PA, USA) for protein extraction.

Filamentous fungi grown in liquid SAB medium were centrifuged at 13,000× *g* for 10 min, then the pellet was washed 3 times with 1 mL of HPLC water. The pellet was resuspended in a hydro-alcoholic solution (300 μL of HPLC water and 900 μL of absolute ethanol).

The fungal material in the hydro-alcoholic suspension was immediately extracted. Extraction began with a 10 min centrifugation at 13,000× *g*. The dried pellet was resuspended in 25 μL of 70% formic acid (Sigma-Aldrich, Paris, France). Then, 25 μL of 100% acetonitrile (VWR International SAS, Radnor, PA, USA) was added and incubated for 10 min. The sample was centrifuged at 13,000× *g* for 2 min just before being deposited on a target. A 1 μL drop of supernatant was deposited onto a polished steel target plate (MTP 96 target plate polished steel, Bruker Daltonics GmbH, Bremen, Germany) and left to air dry. Each deposit was then covered with 1 μL of matrix solution (In vitro diagnostics (IVD) Matrix α-cyano-4-hydroxycinnamic acid (HCCA)-portioned Bruker Daltonics GmbH, Bremen, Germany). Samples were deposited in 8 replicates for each extract.

We also tested 11 isolates (representing 1 isolate for each species, with 2 isolates for *A. sydowii*, 2 isolates for *A. creber,* and 2 isolates for *A. jensenii*, with 1 clinical and 1 environmental isolate for *A. creber* and *A. jensenii*) by direct transfer (or scraping) of fungal material onto a MALDI plate and addition of formic acid to bypass the extraction protocol explained just before.

#### 2.3.3. Spectral Acquisition

Data acquisition was carried out according to Bruker recommendations (MALDI Biotyper: creation of MSP Protocol V1.4, Janvier 2021). Spectra acquisition was performed with Microflex LT/SH MALDI-TOF MS (Bruker Daltonics GmbH, Bremen, Germany) using linear positive mode with mass range *m*/*z* 2000 to 20,000, and an acceleration voltage of 20 kV by the AutoXecute function of Flexcontrol v3.4 software (Bruker Daltonics GmbH, Bremen, Germany) with the “MBT_Autox” method (accumulation = 240 shots). The calibration of the MALDI-TOF MS was automatically performed using a bacterial test standard (BTS) deposit (Bruker Daltonics GmbH, Bremen, Germany) for mass calibration according to the manufacturer’s instructions. Spectra were acquired 3 times on each deposit, resulting in 24 spectra by the extract. 

#### 2.3.4. Quality Control

Data were imported and analyzed using flexAnalysis software v3.4 (Bruker Daltonics GmbH, Bremen, Germany). All spectra were represented with baseline subtraction, smoothing, and automatic scaling on the y-axis. First, the mass accuracy of the BTS was checked (mass deviation < 300 ppm for each peak). Then, the 24 spectra were checked to meet 2 objectives between 6000 and 7000 *m*/*z* to eliminate outliers’ peaks or flatline spectra, and to ensure that the mass deviation between the smallest mass spectrum and the largest mass spectrum was a maximum of 500 ppm. The supplier recommends a minimum of 20 out of 24 spectra that meet the quality requirements described above.

#### 2.3.5. Creation of MSP

Each MSP was created from a minimum of 20 spectra of the same extract using the “MSP creation” function of MaldiBioTyper Compass Explorer^®^ v4.1 software with standard parameters (maximum mass error for each raw spectrum, 2000 Da; desired mass error for the MSP, 200 Da; minimum desired frequency for a peak, 25%; maximum number of peaks for MSP creation, 70). The MSP created was then compared with the Bruker database to avoid any risk of contamination or errors in molecular or phenotypic identification. When a MSP was created, we attempted to obtain a second MSP with two additional days of incubation to obtain MSPs with both young and mature colonies for each of these isolates. The MSPs created were used to generate a new local database named “Fungi CAEN”.

### 2.4. Evaluation of MSP Library Performance

The 30 isolates were tested using the extraction protocol for filamentous fungi from the manufacturer’s instructions. The spectra generated were compared with the two combined databases (Bruker-Daltonics MALDI Biotyper Filamentous Fungi (V4, August 2021) containing 856 MSPs and “Fungi CAEN” containing 59 MSPs). We used the Bruker user manual to interpret the logarithmic score results: high-confidence identification (≥2.00), low-confidence identification (1.70–1.99), and no possible organism identification (<1.70).

We also used three clinical isolates, firstly identified as *A. versicolor* using the “Filamentous Fungi” Bruker database, which were then identified using PCR sequencing as described above and tested against our local database in order to evaluate its performance because these isolates were not included in our local database.

## 3. Results

### 3.1. Interest of Culture Media

Regarding fungal growth on the SAB medium and its cost-effectiveness for spectrometric identification, there were 11 failures in the first and second attempts to create MSPs, probably due to prolonged incubation time in the incubator (Table 2). We observed that the optimum incubation time on the SAB medium for extracting these fungi was two days for *A. amoenus*, *A. fructus*, and *A. sydowii*, three days for *A. protuberus* and *A. creber*, between four and seven days for *A. jensenii*, five days for *A. puulaauensis*, and seven days for *A. tabacinus*. It should be noted that MSPs were easier to obtain for fast-growing species, such as *A. sydowii*, whereas slower-growing species yielded poor results in terms of peptide extraction.

On the MEA medium, there were nine failures in the first attempt to create MSPs and eight failures in the second (Table 2). There was no third attempt for this medium due to a lack of time to reconduct them manually. Incubation times were longer on average, with three days for *A. amoenus*, *A. fructus*, and *A. sydowii*, between eight and nine days for *A. jensenii*, between nine and twelve days for *A. creber*, and up to fourteen days for *A. puulaauensis*. There were not enough spectra for *A. protuberus* to be included in the database on this culture medium (Table 2). 

We observed only failures on the CZA medium. Finally, we tested the liquid SAB medium for the isolates most difficult to extract from SAB, MEA, and CZA media (no MSP obtained or only one from SAB medium): three MSPs were generated from the eight extracted isolates tested (two *A. creber* isolates and one *A. puulaauensis* isolate) (Table 2). The easiest species to extract was *A. sydowii*, as it could be collected more easily than the others (high conidiogenesis). Furthermore, *A. sydowii*, *A. amoenus*, and *A. fructus* grew faster than the others and had a less dry appearance than those that remained longer in the incubator, resulting in a better yield of spectra. The success rate on the first trial was higher with MEA (21/30, 70%) than with the SAB medium (19/30, 63%), but the incubation period was longer. Extraction works better on young colonies regardless of the type of medium. The onset of growth may vary in duration depending on the culture media (longer on MEA than on SAB). However, if we ensured prompt handling from the early stages of growth on the SAB medium, we achieved an excellent success rate on the third attempt (11/11, 100%).

### 3.2. Characteristics of the Local Database

The thirty isolates, from eight different *Aspergillus* species of the *Versicolores* series, were used to create the local spectrometric database, generating fifty-nine MSPs: three for *A. amoenus*, nineteen for *A. creber*, two for *A. fructus*, six for *A. jensenii*, one for *A. protuberus*, three for *A. puulaauensis*, twenty-three for *A. sydowii*, and two for *A. tabacinus*. We were able to create MSPs with both young and mature colonies for only four isolates (two *A. sydowii*, one *A. creber*, and one *A. amoenus*) (Table 2).

Of the 1416 spectra obtained from these 59 MSPs, 130 were eliminated during a visual analysis of the identification spectra due to insufficient quality or obvious dissimilarity with the other replicates. We were able to obtain a MSP from mature colonies for only four isolates (i.e., 4/30, 13% of isolates): two *A. sydowii* isolates on an SAB medium (*A. sydowii* lineage), and *A. amoenus* and *A. creber* on an MEA medium (*A. creber* lineage). Thus, there were two MSPs in the database for those species on SAB and MEA medium (Table 2). For all other species, no conclusive results were obtained from mature colonies (difficult to collect due to low metabolism). In total, we obtained 32 MSPs from the SAB medium, 24 MSPs from the MEA medium, 0 MSPs from the CZA medium, and 3 MSPs from the SAB liquid (Table 2).

**Figure 1:**
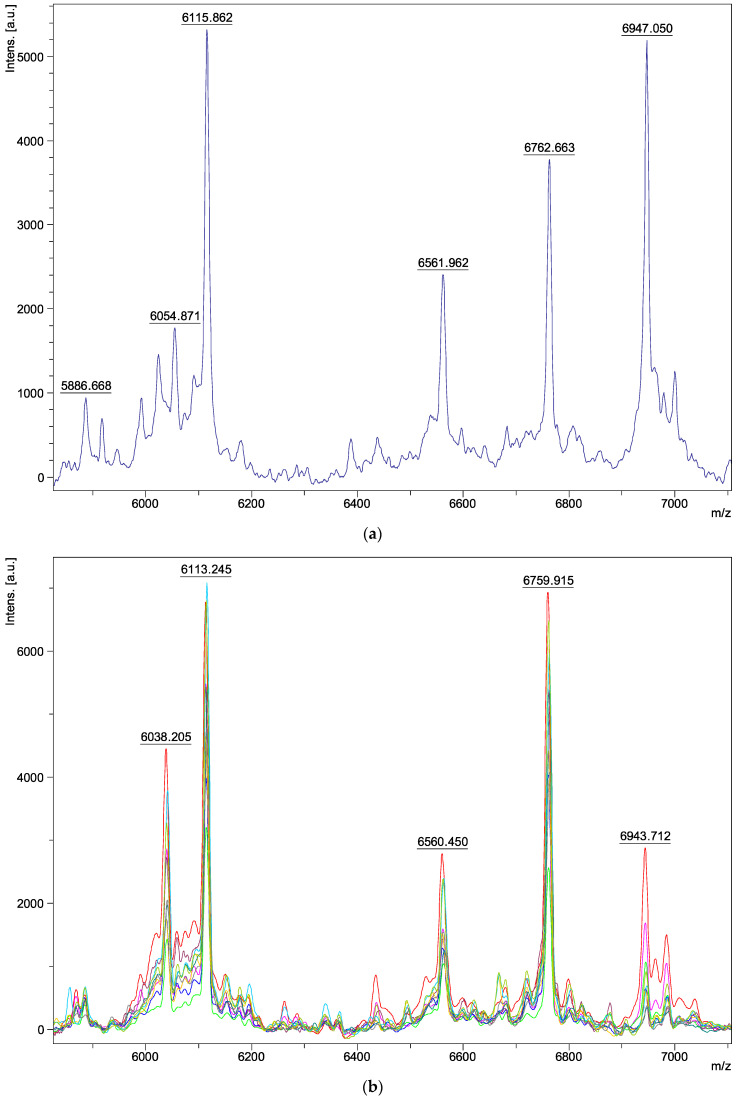

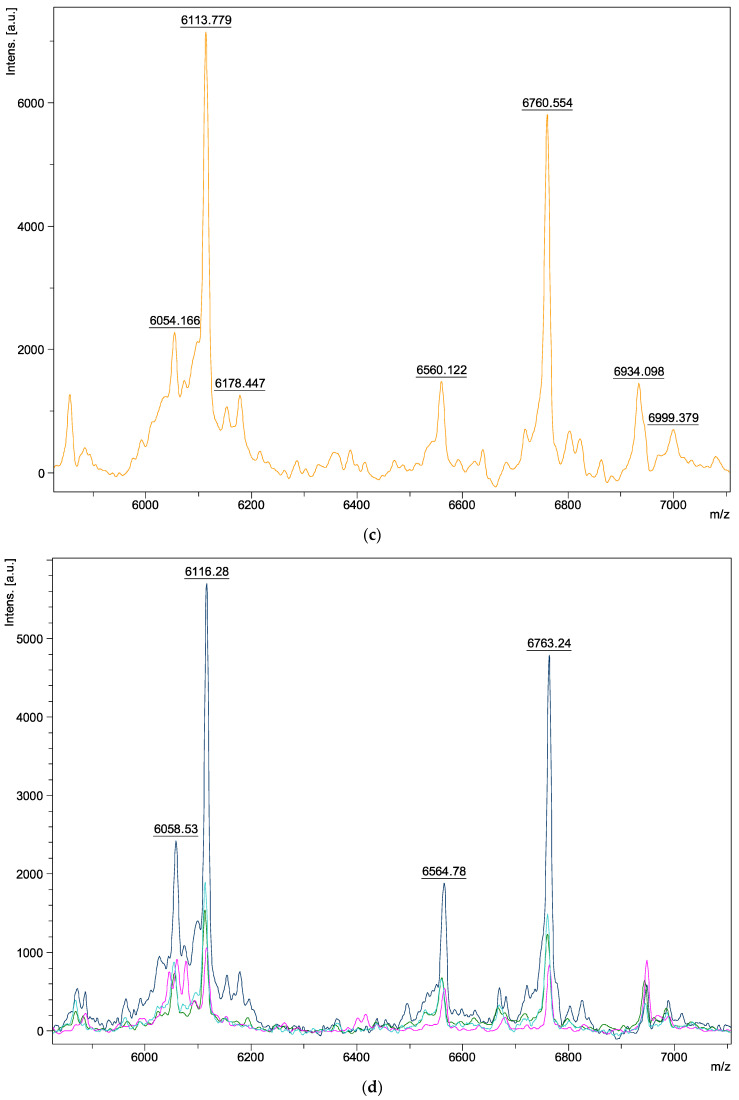

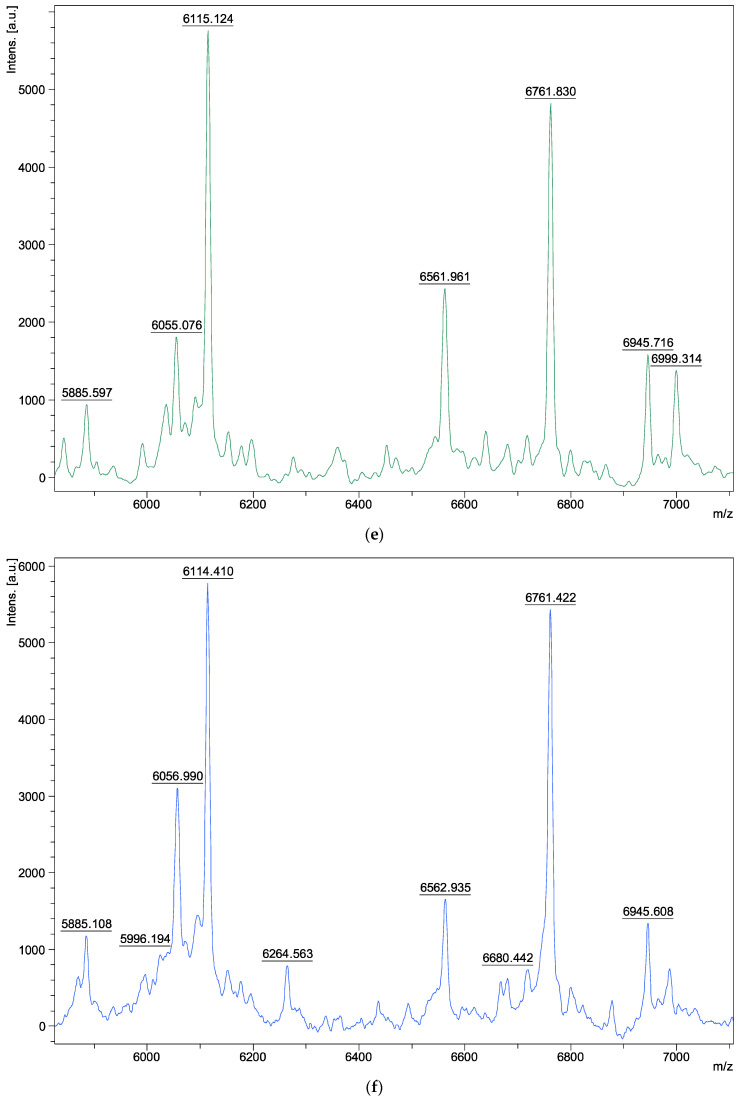

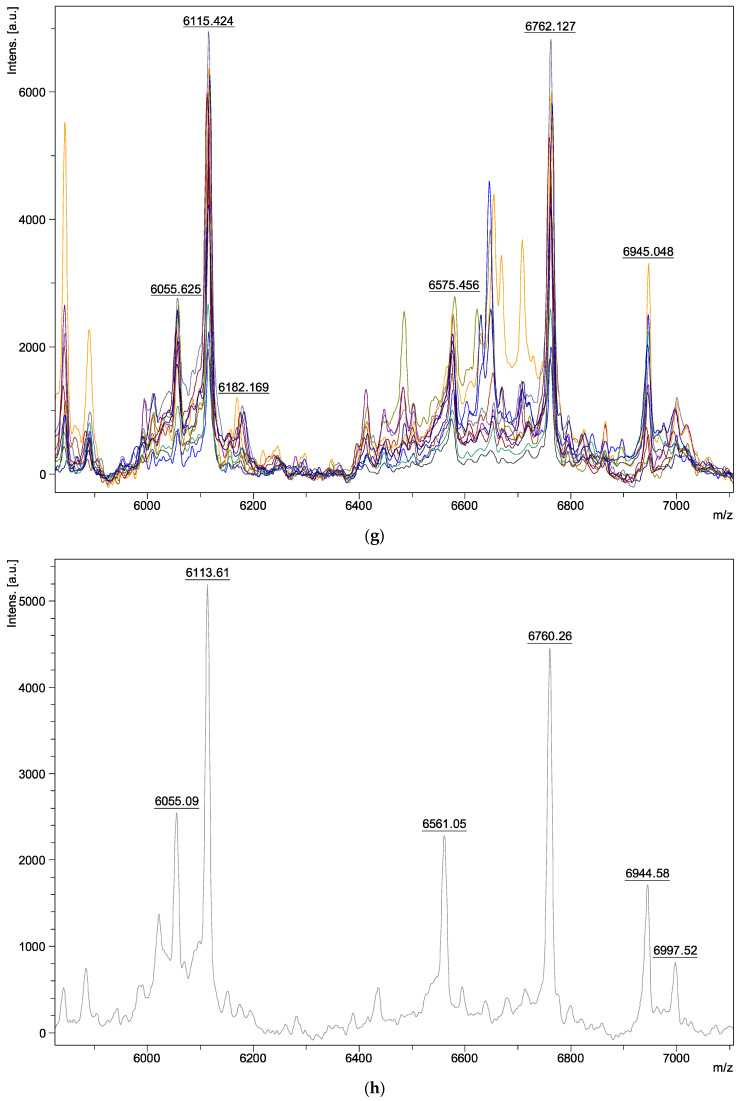

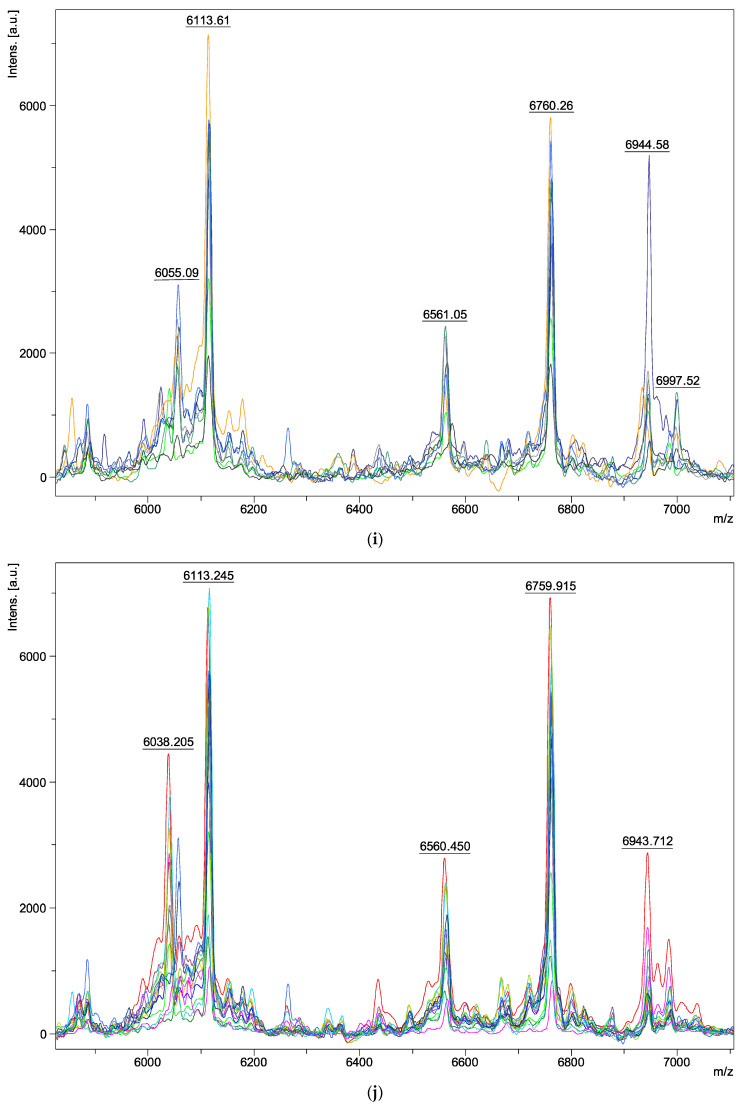

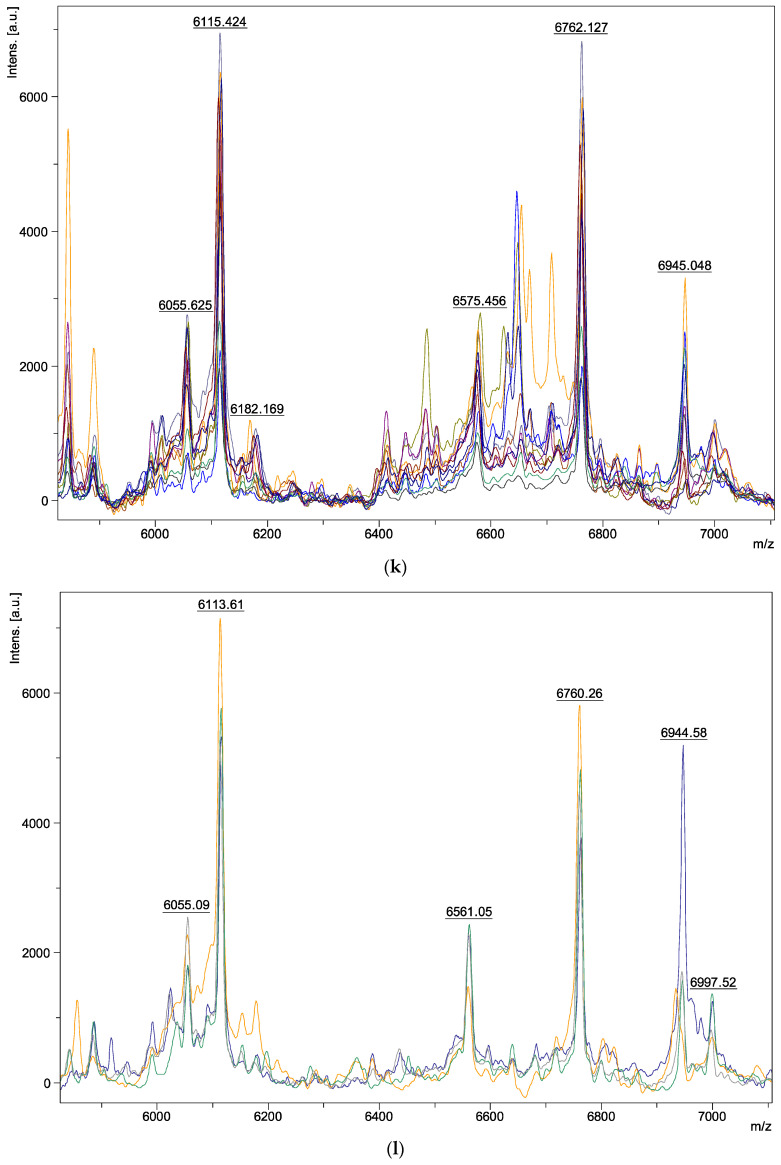
Spectrum of different species of *Aspergillus* series *Versicolores* on SAB between 6000 and 7000 *m*/*z:* (**a**) *A. amoenus N* = 1; (**b**) *A. creber N* = 10; (**c**) *A. fructus N* = 1; (**d**) *A. jensenii N* = 4; (**e**) *A. protuberus N* = 1; (**f**) *A. puulaauensis N* = 1; (**g**) *A sydowii N* = 11; (**h**) *A tabacinus N* = 1; (**i**) 1 isolate of each species; (**j**) *A. creber* lineage *N* = 15; (**k**) *A. sydowii* lineage *N* = 11; (**l**) *A. versicolor* lineage *N* = 4. *N*, number of isolates for each species (**a**–**h**) and for each lineage (**j**–**l**).

**Figure 2:**
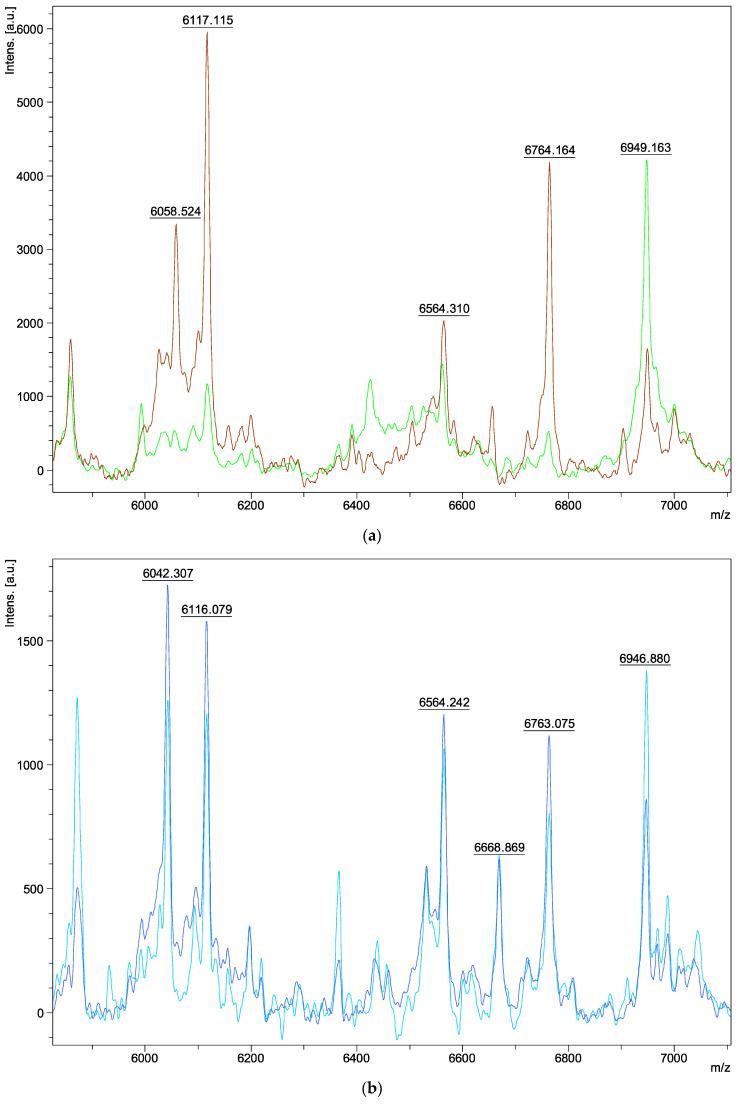

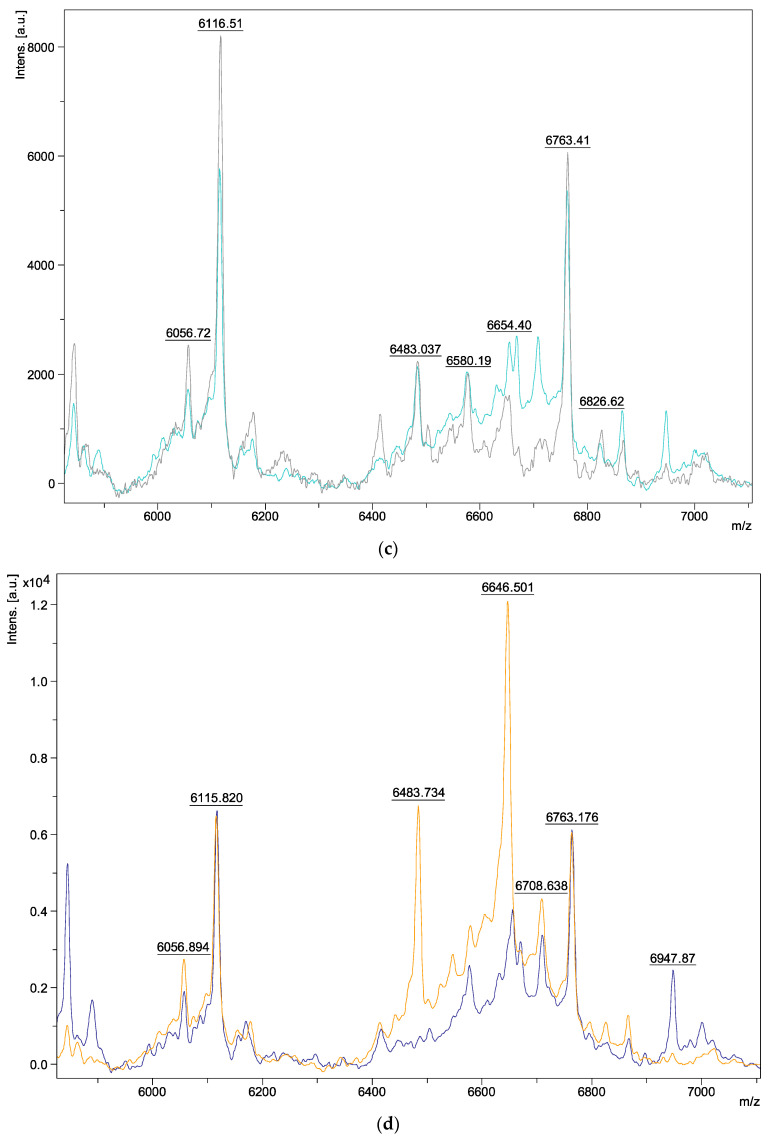
Comparison of spectrum between young and mature colonies of different species of *Aspergillus* series *Versicolores* between 6000 and 7000 *m*/*z*: (**a**) *A. amoenus* 1 (E) on MEA medium (green: young colonies; brown: mature colonies); (**b**) *A. creber* 10 (E) on MEA medium (light blue: young colonies; dark blue: mature colonies); (**c**) *A. sydowii* 19 (C) on SAB medium (grey: young colonies; blue: mature colonies); (**d**) *A. sydowii* 23 (C) on SAB medium (blue: young colonies; orange: mature colonies).

**Figure 3:**
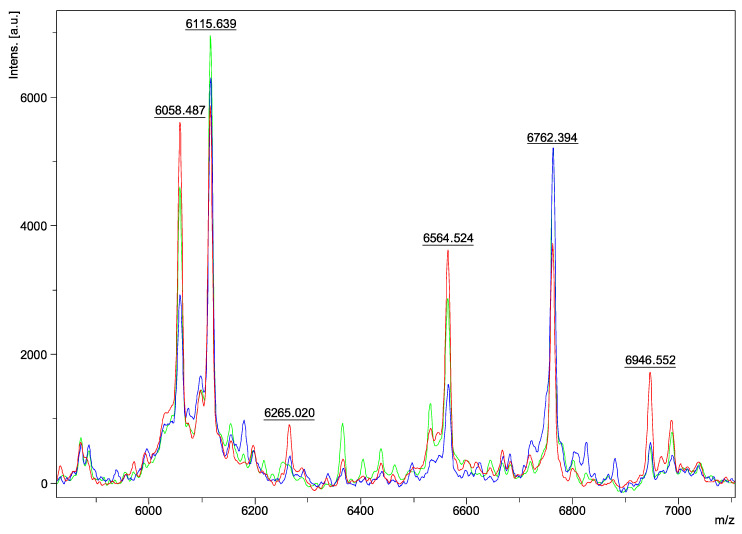
Spectrum of *Aspergillus puulaauensis* on different mediums between 6000 and 7000 *m*/*z* (green = SAB liquid; blue = SAB; red = MEA).

Regarding the characteristics of the spectra, we obtained peaks ranging from 3 to 15 kDa in molecular weight. The analysis was carried out between 6000 and 7000 *m*/*z*, as the most characteristic and intense ions of the fungal macromolecule profile were detected in this range. Spectra within the same species appeared identical (Figure 1a–h). Two peaks, at 6115 ± 2 and 6762 ± 2 *m*/*z*, were found in all isolates belonging to the *Aspergillus* series *Versicolores* (Figure 1i). However, variations were observed in some peaks between the different species: three peaks differed at 6039–6058, 6562–6575, and 6934–6949 m/z depending on the species analyzed (Figure 1i). There were no visible spectral differences between clinical and environmental isolates of the same species (Figure 1a–h) and between young and mature colonies (Figure 2a–d). Furthermore, no spectral differences were detected within the same isolate, depending on the medium used for its extraction (SAB, MEA, liquid SAB) (example of *A. puulaauensis* isolate in Figure 3). 

### 3.3. Evaluation of the Local Database Performance and Comparison with the Bruker Database

To verify our database, each MSP created was post-analyzed against the Bruker and the local databases. Regarding the latter, species identification was correct for 100% of the clinical and environmental isolates extracted. A total of 97% of isolates (29/30) were correctly identified with a log score ≥2.00 (Table 3). Only one isolate (isolate 28) was identified with a score of 1.98 as *A. versicolor* with the Bruker database and as *A. sydowii* with a score of 1.97 with our database. It should be noted that other species were also identified with a log score ≥2.00 (Table 3). Species with only one isolate, such as *A. amoenus*, *A. fructus*, *A. protuberus*, or *A. tabacinus* from the *A. versicolor* lineage of the 2022 taxonomic revision, matched with several species of this lineage due to the absence of MSPs in the database. However, isolates of *A. fructus*, *A. protuberus*, and *A. tabacinus* matched also with other lineages such as *A. sydowii*, *A. puulaauensis,* and *A. creber*, but with a log score <2.00. For the *A. creber* lineage, *A. creber* isolates mainly matched with *A. creber* and sometimes with *A. puulaauensis,* which belong to the same lineage; *A. jensenii* and *A. puulaauensis* isolates matched with all three species of the *A. creber* lineage. For the *A. sydowii* lineage, all isolates matched with *A. sydowii* except for one (isolate 27), which matched once with *A. amoenus* (with a log score ≥2.00), which does not belong to the same lineage. If we consider all the identification profiles of the 30 isolates (30 × 10 = 300), 273 were associated with the local database and 27 with the Bruker database. According to the 2022 taxonomic revision, the “Fungi CAEN” local database showed 97.1% correct identification at the lineage level (265/273) and 78.8% (215/273) at the species level, with a log score ≥1.70. With a log score ≥2.00, better results were obtained, with a correct match of 99.6% (224/225) at the lineage level and 82.7% (186/225) at the species level (Table 3).

Concerning the performance of the Bruker database in relation to the thirty isolates tested, *A. versicolor* 120227_14 ETL was identified for *A. amoenus*, *A. fructus*, *A. protuberus*, and *A. tabacinus*; *A. versicolor* 392 UGB and *A. versicolor* D_16_256_8_1 LLH for *A. amoenus* and *A.fructus*; *A. versicolor* 1343 MPA for *A. amoenus*, *A. fructus*, and *A. sydowii* (six times); *A. versicolor* F51 LLH for *A. creber* (six times) and *A. puulaauensis*; *A. versicolor* DSM 63292 DSM only for *A. creber*; and *A. sydowii* 2008_141783 MUZ and *A. versicolor* 2009_137364 MUZ only for *A. sydowii* (Table 3). Thus, the “Filamentous Fungi” Bruker database showed a correct identification of 44.4% (12/27) at the lineage level (corresponding only to *A. versicolor* and *A. sydowii* lineages) and 0.7% (2/27) at the species level (corresponding only to *A. versicolor* and *A. sydowii* species), with a log score ≥1.70. With a log score ≥2.00, the correct identification rate decreased to 33.3% (6/18) at the lineage level and 0% (0/18) at the species level (Table 3).

The *Aspergillus versicolor* strains in the Bruker database did not always correspond to the *A. versicolor* species, according to the new taxonomic revisions. It is difficult to know to which species exactly these reference strains can be identified; it is likely *that A. versicolor* F51 LLH and *A. versicolor* DSM 63292 DSM are actually *A. creber,* and that *A. versicolor* 2009_137364 MUZ is *A. sydowii*. On the other hand, *A. sydowii* 2008_141783 MUZ appears to be well identified. For the other strains, there are not enough spectra to make a comparison (Table 3).

To speed up turnaround times in laboratory routine, we wanted to find out whether the simplified extraction method provided by Bruker (i.e., direct transfer of fungal material onto the MALDI plate and addition of formic acid) resulted in the correct identification of *Aspergillus* isolates of the *Versicolores* series. The results of this technique for eleven isolates were highly inconsistent and only worked with one isolate (isolate 30), with a log score ≥2.00: *A. versicolor* 120227_14 ETL (2.31), *A. versicolor* D_16_256_8_1 LLH (2.15), and *A. versicolor* 392 UGB (2.04) from the Bruker database and *A. tabacinus* (2.30) from the local database. For one isolate (isolate 21), all ten profiles were below the organism identification threshold (<1.70) but matched with *A. sydowii* from the local database (seven profiles) and *A. versicolor* 2009_137364 MUZ, *A. versicolor* 1343 MPA, and *A. sydowii* 2008_141783 MUZ from the Bruker database (three profiles). No spectrum was detected for the remaining nine isolates.

In addition, three clinical isolates previously identified as *A. versicolor* in the Bruker database and then identified by PCR sequencing as *A. sydowii*, *A. tabacinus,* and *A. creber*, were tested in our local database. *A. sydowii* matched 100% to *A. sydowii* from the “Fungi CAEN” database with a score ≥2.00 (four profiles ≥ 2.00 and six profiles between 1.70 and 2.00). *A. tabacinus* matched with *A. tabacinus* (1.97), *A. fructus* (1.92 and 1.76), *A. protuberus* (1.90), and *A. amoenus* (1.84) from our “Fungi CAEN” database; and *A. versicolor* 120227_14 ETL (2.26), *A. versicolor* 392 UGB (1.98), *A. versicolor* D_16_256_8_1 LLH (1.93), *A. versicolor* DSM 63292 DSM (1.77), and *A. sydowii* F68 RLH (1.88) from the Bruker database. *A. creber* matched to *A. creber* (seven profiles ≥ 2.00 and one profile between 1.70 and 2.00) from “Fungi CAEN” and *A. versicolor* F51 LLH (2.18) and *A. versicolor* DSM 63292 DSM (1.99) from the Bruker database. Finally, the “Fungi CAEN” local database showed 100% correct identification at the lineage level (23/23) and 82.6% (19/23) at the species level, with a log score ≥ 1.70, whereas the Bruker database showed a correct identification of 57.1% at the lineage level (4/7) and 0% (0/7) at the species level, with a log score ≥1.70. 

## 4. Discussion

Many *Aspergillus* species of the *Versicolores* series are closely related and share similar morphological and molecular characteristics [17,18]. This can make accurate identification challenging using traditional methods. Molecular techniques, on the other hand, are expensive, time-consuming, and poorly suited to laboratory routines. MALDI-TOF MS has proven to be a valuable tool for differentiating species on the basis of their unique protein profiles. The high discriminatory power of MALDI-TOF MS, combined with its low cost and speed, is particularly important for the identification of closely related species, as even small differences in protein profiles can distinguish them [28].

Several studies have shown that MALDI-TOF MS can be successfully used to identify fungi in this series. Recently, Reeve et al. used MALDI-TOF MS technology to characterize 40 environmental isolates of *Aspergillus* series *Versicolores* [29]. They found consistent identifications with the Bruker database, identifying *A. versicolor* 2009_137364 MUZ *and A. versicolor* F51 LLH, which did not belong to the same spectral-linkage groups. In our database, *A. versicolor* 2009_137364 MUZ and *A. versicolor* F51 LLH matched with *A. sydowii* and *A. creber*, respectively. Masih et al. evaluated the performance of MALDI-TOF MS for the identification of different and rare *Aspergillus*, including *A. versicolor* and *A. sydowii* [30]. The Bruker database identified only eight (38%) of the twenty-three rare *Aspergillus* isolates compared with 95% for their local mycology database. Alanio et al. demonstrated the usefulness of MALDI-TOF MS for the identification of different *Aspergillus* species: 138 out of 140 (98.6%) were correctly identified. Two atypical isolates could not be identified, but no isolate was misidentified (specificity: 100%) [31]. It should be noted that the sensitivity of MALDI-TOF MS varies depending on the *Aspergillus* species. According to the study by Shao et al., MALDI-TOF MS performed well in identifying species of the *Aspergillus Fumigati* and *Terrei* sections, but performed poorly in distinguishing certain closely related species of the *Nigri*, *Flavi*, and *Nidulantes* sections, which include *Aspergillus* of the *Versicolores* series [24].

Another study by Vidal-Aguiar et al. compared the use of the MALDI-TOF MS with other molecular characterization methods, such as DNA sequencing, to identify different *Aspergillus* species [22]. The MALDI-TOF MS was found to have similar accuracy to the DNA sequencing method with no discordant identifications, but was much faster and less expensive. It is important to note that while this study focused on *Aspergillus* sections *Fumigati, Flavi*, *Nigri,* and *Terrei*, it also included *Aspergillus* series *Versicolores*, with the species *A. creber*, *A. sydowii,* and *A. tabacinus* each represented by one isolate. Species identification was obtained for one hundred and ninety-one isolates (95.5%), and only nine (4.5%) could not be identified at the species level, grown on solid or liquid media [22].

We have created the very first local MALDI-TOF MS database for the different species of *Aspergillus* series *Versicolores* from French clinical and environmental isolates. Following the new taxonomic classification system for *Aspergillus* series *Versicolores,* published by Sklenář et al. in 2022, our results correspond perfectly to this new taxonomy with a correct identification rate of 97.1% at the lineage level compared to the “Filamentous Fungi” Bruker database, which achieved a correct identification of only 44.4%. Despite the lack of isolates for some species, we managed to identify the main lineages of this new taxonomy (*A. sydowii*, *A. creber*, and *A. versicolor*) [17]. As far as supplier databases are concerned, it would be wise to rename the reference spectra “*Aspergillus* series *Versicolores*” instead of “*Aspergillus versicolor*”, until the taxonomic revision has been integrated. 

Nevertheless, our local database still has some limitations. First, some species were rarely found in clinical or environmental samples, and were therefore represented by a single isolate: *A. amoenus*, *A. fructus*, *A. protuberus*, *A. puulaauensis*, and *A. tabacinus*. All these isolates belong to the *A. versicolor* lineage in the new 2022 taxonomy, except *A. puulaauensis*, which belongs to the *A. creber* lineage. The isolates tested in this study come exclusively from France, particularly Normandy, and some species were absent from our database, notably the *A. subversicolor* lineage in the new 2022 taxonomy. Given the numerous samples in different biotopes over time in this geographical area (indoor air of different homes, hospital air, agricultural environment, and human clinical samples), we can still conclude that our collection of *Aspergillus* series *Versicolores* is representative of the local fungal ecology [18]. Five species were represented by a single isolate and nine species were not present in our database, which may explain the lack of intraspecific variability in the database and why the MALDI-TOF MS technique may not be able to differentiate them from other species [17,32]. Therefore, it will be necessary to include additional isolates belonging to these species or new species when they become available in order to expand the database and improve its reliability.

Only four MSPs were created from mature colonies (two *A. sydowii*, one *A. creber*, and one *A. amoenus*). As their spectra show no differences, it is unnecessary to generate spectra at variable growth times for these species. The method recommended by Bruker for the identification of fungi is the full-extraction protocol. Some studies have also optimized this protocol to improve sensitivity [33,34], while some research teams have suggested the utilization of alternate matrix types, including sinapinic acid or 3,4-dihydroxycinnamic acid [20]. Even if it is difficult to change the matrix in a medical diagnostic laboratory subject to accreditation, these proposals are promising. We tested direct transfer, but it was not as effective as the full-extraction protocol. However, it may be interesting in the future to implement an identification protocol using a rapid method, similar to that used for yeasts [34]. 

Fungal growth conditions have a significant impact on the variability and quality of spectra, which are affected by culture media and incubation time. The media we used have not been validated by Bruker, which could explain the numerous failures on MEA and CZA media, where colonies were drier, whereas the SAB medium seemed to perform better [35].

To conclude, the use of MALDI-TOF MS for the identification of *Aspergillus* series *Versicolores* has certain limitations. The first is the intraspecific variability of these fungi, which can lead to overlapping spectra between closely related species, as shown in the study by Becker et al. [32]. Our database is therefore effective for complete identification of the lineage according to the latest taxonomic revision, but not of the species. Another limitation is the lack of reference spectra or misidentified reference spectra in commercial databases for some species within *Aspergillus* series *Versicolores*. This can result in misidentification or no identification, which may require additional confirmatory tests, such as DNA sequencing [22,23,24]. Finally, the accuracy of MALDI-TOF MS can be affected by the quality of sample preparation, age of culture, and growing conditions [35].

## 5. Conclusions

The taxonomic history of *Aspergillus* series *Versicolores* reflects the evolution of the means used to better characterize it. The use of multiple lines of evidence, including morphological, molecular, and chemical data, has led to a more nuanced understanding of the diversity within this group of fungi, resulting in the four lineages proposed in the last taxonomic revision of 2022 [17]. MALDI-TOF MS represents a reliable and efficient method for the identification of *Aspergillus* series *Versicolores*. This technology can improve the speed and accuracy of routine fungal identification in microbiology laboratories. The implementation and availability of this database, through its possible inclusion in the update of the supplier’s database, will help improve microbiological diagnosis and assess the exact role of these species in an environmental and clinical context.

## Figures and Tables

**Table 1:** List and origin of *Aspergillus* series *Versicolores* isolates.

Species	Isolate Number	Origins
*Aspergillus amoenus*	1 (E)	Mold-damaged home
*Aspergillus creber*	2 (E)	Cancer treatment center
	3 (E)	Cancer treatment center
	4 (E)	*Serpula lacrymans*-damaged home
	5 (C)	Skin scales of right foot
	6 (C)	Skin scales of right foot
	7 (C)	Sputum
	8 (E)	Mold-damaged home
	9 (E)	Mold-damaged home
	10 (E)	Mold-damaged home
	11 (E)	Mold-damaged home
*Aspergillus fructus*	12 (C)	Nail of big toe
*Aspergillus jensenii*	13 (C)	Scalp
	14 (C)	Bronchoalveolar lavage fluid
	15 (C)	Bronchoalveolar lavage fluid
	16 (E)	Mold-damaged home
*Aspergillus protuberus*	17 (E)	Cancer treatment center
*Aspergillus puulaauensis*	18 (C)	Armpit skin
*Aspergillus sydowii*	19 (C)	Bronchoalveolar lavage fluid
	20 (C)	Bronchoalveolar lavage fluid
	21 (C)	External auditory canal
	22 (C)	Bronchoalveolar lavage fluid
	23 (C)	Bronchoalveolar lavage fluid
	24 (C)	Bronchoalveolar lavage fluid
	25 (C)	Bronchoalveolar lavage fluid
	26 (C)	Nail
	27 (C)	Bronchoalveolar lavage fluid
	28 (C)	Bronchoalveolar lavage fluid
	29 (C)	Bronchoalveolar lavage fluid
*Aspergillus tabacinus*	30 (C)	Sputum (cystic fibrosis)

E, environmental isolate; C, clinical isolate.

**Table 2:** Results of 59 MSPs of *Aspergillus* series *Versicolores* on different culture media.

Species	Isolate Number	Culture Medium							Number of MSPs Obtained
		SAB			MEA		CZA	LIQUID SAB	
		First Attempt	Second Attempt	Third Attempt	First Attempt	Second Attempt	First Attempt	First Attempt	
*Aspergillus amoenus*	1 I	1 MSP	/	/	2 MSPs	/	0 MSP	/	3
*Aspergillus creber*	2 (E)	0 MSP	0 MSP	1 MSP	0 MSP	0 MSP	0 MSP	1 MSP	2
	3 (E)	0 MSP	0 MSP	1 MSP	1 MSP	/	/	/	2
	4 (E)	0 MSP	0 MSP	1 MSP	0 MSP	0 MSP	0 MSP	1 MSP	2
	5 (C)	0 MSP	0 MSP	1 MSP	1 MSP	/	/	/	2
	6 (C)	1 MSP	/	/	0 MSP	0 MSP	0 MSP	0 MSP	1
	7 (C)	0 MSP	0 MSP	1 MSP	0 MSP	1 MSP	0 MSP	/	2
	8 (E)	1 MSP	/	/	0 MSP	0 MSP	0 MSP	0 MSP	1
	9 (E)	0 MSP	0 MSP	1 MSP	1 MSP	/	/	/	2
	10 (E)	1 MSP	/	/	2 MSPs	/	/	/	3
	11 (E)	0 MSP	0 MSP	1 MSP	1 MSP	/	/	/	2
*Aspergillus fructus*	12 (C)	1 MSP	/	/	1 MSP	/	0 MSP	/	2
*Aspergillus jensenii*	13 (C)	1 MSP	/	/	1 MSP	/	/	/	2
	14 (C)	0 MSP	0 MSP	1 MSP	0 MSP	0 MSP	0 MSP	0 MSP	1
	15 (C)	1 MSP	/	/	1 MSP	/	/	/	2
	16 (E)	0 MSP	0 MSP	1 MSP	0 MSP	0 MSP	0 MSP	0 MSP	1
*Aspergillus protuberus*	17 (E)	0 MSP	0 MSP	1 MSP	0 MSP	0 MSP	0 MSP	0 MSP	1
*Aspergillus puulaauensis*	18 (C)	0 MSP	0 MSP	1 MSP	1 MSP	/	0 MSP	1 MSP	3
*Aspergillus sydowii*	19 (C)	2 MSPs	/	/	1 MSP	/	/	/	3
	20 (C)	1 MSP	/	/	1 MSP	/	/	/	2
	21 (C)	1 MSP	/	/	1 MSP	/	/	/	2
	22 (C)	1 MSP	/	/	1 MSP	/	/	/	2
	23 (C)	2 MSPs	/	/	0 MSP	0 MSP	0 MSP	/	2
	24 (C)	1 MSP	/	/	1 MSP	/	/	/	2
	25 (C)	1 MSP	/	/	1 MSP	/	/	/	2
	26 (C)	1 MSP	/	/	1 MSP	/	/	/	2
	27 (C)	1 MSP	/	/	1 MSP	/	/	/	2
	28 (C)	1 MSP	/	/	1 MSP	/	/	/	2
	29 (C)	1 MSP	/	/	1 MSP	/	/	/	2
*Aspergillus tabacinus*	30 (C)	1 MSP	/	/	1 MSP	/	0 MSP	/	2

E, environmental isolate; C, clinical isolate; SAB, Sabouraud-chloramphenicol-gentamicin agar; MEA, malt extract agar; CZA, CZAPEK agar. 0 MSP = Failed attempt, 1 MSP = obtained from a young colony, 2 MSPs = obtained from young and mature colonies. /, not performed because MSP was already obtained during the previous attempt.

**Table 3:** Comparison of the Bruker database (Filamentous Fungi) and the local database (“Fungi CAEN”).

Species	Isolates Number	Bruker Database				Local Database				Total Number of Profiles (/10)	
		Log Score ≥ 2.00	Number of Profiles	1.70 ≤ Log Score < 2.00	Number of Profiles	Log Score ≥ 2.00	Number of Profiles	1,70 ≤ Log Score < 2.00	Number of Profiles		
*Aspergillus amoenus*	1 (E)	*Aspergillus versicolor* 120227_14 ETL	1		0	*Aspergillus amoenus*	2		0		10
		*Aspergillus versicolor* 392 UGB	1			*Aspergillus tabacinus*	1				
		*Aspergillus versicolor* D_16_256_8_1 LLH	1			*Aspergillus fructus*	2				
		*Aspergillus versicolor* 1343 MPA	1			*Aspergillus protuberus*	1				
*Aspergillus creber*	2 (E)	*Aspergillus versicolor* F51 LLH	1		0	*Aspergillus creber*	7		0		10
		*Aspergillus versicolor* DSM 63292 DSM	1			*Aspergillus puulaauensis*	1				
	3 (E)		0		0	*Aspergillus creber*	9		0		10
						*Aspergillus puulaauensis*	1				
	4 (E)	*Aspergillus versicolor* F51 LLH	1		0	*Aspergillus creber*	8		0		10
						*Aspergillus puulaauensis*	1				
	5 (C)		0		0	*Aspergillus creber*	9		0		10
						*Aspergillus puulaauensis*	1				
	6 (C)	*Aspergillus versicolor* F51 LLH	1		0	*Aspergillus creber*	8		0		10
						*Aspergillus puulaauensis*	1				
	7 (C)		0		0	*Aspergillus creber*	10		0		10
	8 (E)	*Aspergillus versicolor* F51 LLH	1		0	*Aspergillus creber*	9		0		10
	9 (E)	*Aspergillus versicolor* F51 LLH	1		0	*Aspergillus creber*	9		0		10
	10 (E)		0		0	*Aspergillus creber*	10		0		10
	11 (E)	*Aspergillus versicolor* F51 LLH	1		0	*Aspergillus creber*	8		0		10
						*Aspergillus puulaauensis*	1				
*Aspergillus fructus*	12 (C)	*Aspergillus versicolor* 120227_14 ETL	1	*Aspergillus versicolor* 1343 MPA	1	*Aspergillus fructus*	2	*Aspergillus protuberus*	1		10
				*Aspergillus versicolor* 392 UGB	1	*Aspergillus amoenus*	1	*Aspergillus puulaauensis*	1		
				*Aspergillus versicolor* D_16_256_8_1 LLH	1	*Aspergillus tabacinus*	1				
*Aspergillus jensenii*	13 (C)		0		0	*Aspergillus jensenii*	4		0		10
						*Aspergillus creber*	3				
						*Aspergillus puulaauensis*	3				
	14 (C)		0		0	*Aspergillus jensenii*	3	*Aspergillus jensenii*	1		10
						*Aspergillus creber*	2	*Aspergillus creber*	3		
								*Aspergillus puulaauensis*	1		
	15 (C)		0		0	*Aspergillus jensenii*	5		0		10
						*Aspergillus creber*	4				
						*Aspergillus puulaauensis*	1				
	16 (E)		0		0	*Aspergillus jensenii*	3	*Aspergillus jensenii*	2		10
						*Aspergillus creber*	2	*Aspergillus creber*	2		
								*Aspergillus puulaauensis*	1		
*Aspergillus protuberus*	17 (E)		0	*Aspergillus versicolor* 120227_14 ETL	1	*Aspergillus protuberus*	1	*Aspergillus fructus*	2		10
						*Aspergillus tabacinus*	1	*Aspergillus sydowii*	3		
						*Aspergillus amoenus*	1	*Aspergillus puulaauensis*	1		
*Aspergillus puulaauensis*	18 (C)	*Aspergillus versicolor* F51 LLH	1		0	*Aspergillus puulaauensis*	3		0		10
						*Aspergillus jensenii*	2				
						*Aspergillus creber*	4				
*Aspergillus sydowii*	19 (C)	*Aspergillus versicolor* 1343 MPA	1		0	*Aspergillus sydowii*	8	*Aspergillus sydowii*	1		10
	20 (C)		0		0	*Aspergillus sydowii*	9	*Aspergillus sydowii*	1		10
	21 (C)		0		0	*Aspergillus sydowii*	9	*Aspergillus sydowii*	1		10
	22 (C)	*Aspergillus versicolor* 1343 MPA	1		0	*Aspergillus sydowii*	6	*Aspergillus sydowii*	3		10
	23 (C)		0	*Aspergillus sydowii* 2008_141783 MUZ	1	*Aspergillus sydowii*	2	*Aspergillus sydowii*	7		10
	24 (C)		0	*Aspergillus versicolor* 1343 MPA	1	*Aspergillus sydowii*	5	*Aspergillus sydowii*	4		10
	25 (C)		0	*Aspergillus sydowii* 2008_141783 MUZ	1	*Aspergillus sydowii*	8	*Aspergillus sydowii*	1		10
	26 (C)		0		0	*Aspergillus sydowii*	10		0		10
	27 (C)	*Aspergillus versicolor* 1343 MPA	1		0	*Aspergillus sydowii*	8		0		10
						*Aspergillus amoenus*	1				
	28 (C)		0	*Aspergillus versicolor* 1343 MPA	1		0	*Aspergillus sydowii*	8		10
				*Aspergillus versicolor* 2009_137364 MUZ	1						
	29 (C)	*Aspergillus versicolor* 1343 MPA	1		0	*Aspergillus sydowii*	9		0		10
*Aspergillus tabacinus*	30 (C)	*Aspergillus versicolor* 120227_14 ETL	1		0	*Aspergillus tabacinus*	2	*Aspergillus fructus*	1		10
						*Aspergillus amoenus*	1	*Aspergillus jensenii*	2		
						*Aspergillus protuberus*	1	*Aspergillus amoenus*	1		
						*Aspergillus fructus*	1				
Total number of profiles			18		9		225		48		300

E, environmental isolate; C, clinical isolate.

## Data Availability

The data of the local database are available from the corresponding author, upon reasonable request.
